# Diagnostic value of quantitative MR spectroscopy metabolite ratios for Canavan disease

**DOI:** 10.3389/fradi.2026.1826170

**Published:** 2026-07-15

**Authors:** Majid Reza Tahsini, Marzena Wylezinska-Arridge, Mohammad Zare, Sasan Saket, Mohammad Aidin Farahvash, Mahdis Morovvati, Elham Rahimian, Sotirios Bisdas

**Affiliations:** 1 Haghighat medical imaging research center, Haghighat medical imaging center, Tehran, Iran; 2 Lysholm Department of Neuroradiology, The National Hospital for Neurology and Neurosurgery, UCL Hospitals NHS Trust, London, United Kingdom; 3 Department of Statistics, Faculty of Mathematical Sciences, Alzahra University, Tehran, Iran; 4 Department of Pediatric Neurology, School of Medicine, Imam Hossein & Mofid Children’s Hospitals, Tehran, Iran; 5 Roozbeh Hospital, Faculty of Psychiatry, Tehran University of Medical Sciences, Tehran, Iran; 6 Department of Translational Neuroscience and Stroke, Queen Square Institute of Neurology, University College London, London, United Kingdom; 7 Department of Neuroradiology and Advanced Neuroimaging, Athens Medical Centre, Athens, Greece; 8 Medical Faculty, European University Cyprus, Nicosia, Cyprus

**Keywords:** Canavan disease, hypomyelinating disorders, leukodystrophies, magnetic resonance spectroscopy, N-acetyl-aspartate (NAA)

## Abstract

**Purpose:**

Canavan disease (CD) is a leukodystrophy marked by deficiency in the aspartoacylase enzyme and accumulation of N-acetyl aspartate (NAA) in the white matter. MRI and MRS are crucial diagnostic tools for CD. MRS shows a distinctive, significant elevation of the NAA peak, which is typically not quantified and thus not objectively assessed as a diagnostic criterion. This study aimed to establish quantitative cutoff values for the elevated NAA peak on proton MR spectroscopy (^1^H-MRS) in patients with CD and hence, incorporate MRS into the diagnostic algorithm.

**Methods:**

A cross-sectional retrospective diagnostic study involving 18 confirmed CD cases and 10 healthy individuals was conducted to assess metabolic profiles and determine a diagnostic cutoff for NAA in CD. 1H MRS (TE: 135 ms) was utilized, and data were post-processed using specialized software to quantify the ratios of the three primary metabolites: NAA, Cho, and Cr.

**Results:**

The analysis of the CD cases demonstrated substantial elevation in NAA levels compared to the standard control group with the NAA/Cr and NAA/Cho ratios significantly increased in CD patients compared to controls (*p* < 0.001 for both). Diagnostic cut-off values of 2.75 for NAA/Cr and 7.18 for NAA/Cho yielded an optimism-corrected AUC = 0.96 (95% CI 0.95–1.00) after bootstrap validation.

**Conclusion:**

This study represents one of the larger single-center cohorts evaluating quantitative MRS metabolite ratios in Canavan disease and proposes preliminary diagnostic thresholds for improved diagnostic objectivity.

## Introduction

CD is an autosomal recessive leukodystrophy caused by a deficiency in the ASPA enzyme. This enzyme metabolizes NAA (1). Subsequently, NAA accumulation and reduced acetyl-CoA availability cause myelin loss and edema. NAA accumulation seems to play an osmoregulatory role in the brain and causes toxic myelin splitting of white matter (2). The result is a progressive spongiform degeneration of brain white matter.

Neuroimaging, especially MRI and MRS, plays a significant role in diagnosing CD phenotyping as in the infancy-onset variant, the subarcuate U-fibers have a particularly early involvement while the periventricular white matter is typically spared (3–5). In the advanced disease stage, MRI shows a diffuse leukoencephalopathy with swelling of the white matter involving in both the supratentorial and infratentorial compartments (6). The affected white matter appears hypointense on T1-weighted hyperintense on T2-weighted images, and shows restricted diffusivity on DWI owing to intramyelinic edema (7). The thalami and globi pallidi are also involved, whereas the caudate nuclei, putamina, and claustrum are typically spared (6, 8).

CD has a pathognomonic appearance on 1H-MRS, characterized by marked, isolated increase of the NAA peak (9). Other nonspecific features include decrease in Cho and Cr, an increase in myo-inositol (mI), and the frequent presence of lactate (10–12). In previous studies, MRS was acquired with an intermediate echo time (TE = 135 ms), which improves spectral resolution, suppresses macromolecule-related baseline distortions and demonstrates less variability in the metabolite estimations across scanners (13–15).

Nonetheless, leukodystrophies with macrocephaly such as Alexander disease, or other leukodystrophies such as metachromatic leukodystrophy (MLD) and Leigh syndrome, can mimic CD in structural MRI. These conditions typically lack the hallmark of markedly elevated NAA peak. Incorporating quantitative metabolite ratio analysis could aid in distinguishing CD from these mimics with the caveat that certain hypomyelinating disorders, such as Pelizaeus–Merzbacher disease (PMD), may also present with elevated NAA peaks (16).

While a prominent NAA peak is often considered pathognomonic for CD, visual interpretation remains subjective and may suffer from interobserver variability. On contrary, robust metabolite ratios and diagnostic cut-off values could enhance diagnostic accuracy.

In clinical practice, the major diagnostic challenge is not distinguishing Canavan disease from normal brain tissue, but rather differentiating it from other leukodystrophies and metabolic disorders with overlapping imaging features. Although elevated NAA is considered a hallmark of Canavan disease, reliance on qualitative visual assessment may lead to diagnostic uncertainty in such cases. Therefore, the establishment of quantitative metabolite ratios and diagnostic thresholds may improve objectivity and reproducibility in clinical decision-making.

## Materials and methods

### Study design and participants

This IRB- and Ethics committee-approved retrospective cross-sectional study included 18 confirmed cases of CD and 10 neurologically normal control subjects, who were retrospectively identified having no neurological or psychiatric history, normal neurodevelopment, and unremarkable MRI and MRS. Clinical, biochemical, and imaging data were extracted from the institutional pediatric neuroimaging archive, comprising more than 2,000 pediatric cases prospectively collected since 2012. CD diagnosis was based on a combination of characteristic leukodystrophy in MRI and confirmatory testing, including ASPA gene mutations, NAA on MRS, and/or increased urinary NAA levels. In this study, all included patients had definitive genetic confirmation of Canavan disease, with variants in the ASPA gene identified by whole exome sequencing and confirmed by Sanger sequencing, while biochemical findings (elevated NAA in urine or blood) were used as supportive evidence. Because the causative variants were unambiguously identified by this two-step approach, additional deletion/duplication analysis was not required.

The control subjects were retrospectively identified from the same institutional pediatric neuroimaging archive. Their MRI/MRS studies had been obtained as part of routine clinical care for indications such as headache or benign neonatal sleep myoclonus, but all were subsequently confirmed to have no neurological or psychiatric history, normal neurodevelopment, and unremarkable MRI and MRS. No healthy volunteers underwent imaging solely for research purposes. “Healthy” status was established by: (1) absence of any neurological or psychiatric diagnosis, (2) normal neurological examination at the time of imaging, (3) normal neurodevelopmental follow-up for at least 12 months post-imaging, and (4) independent visual assessment of MRI and MRS as normal by two pediatric neuroradiologists.

### MRI and MRS protocols

MRI was performed using a 1.5 T Siemens Avanto scanner (Magnetom Syngo MR B17, Siemens Healthcare, Erlangen, Germany) with a 12-element Head Matrix coil. Acquired sequences included T1-weighted (TR/TE 500/8 ms), T2-weighted (TR/TE 4000/106 ms), fluid-attenuated inversion recovery (FLAIR; TR/TE/TI 8000/100/2370 ms), and diffusion-weighted imaging (TR/TE 3300/136 ms). Contrast agent was not used. Sedation or general anesthesia was administered to all participants.

Proton MRS was performed using a two-dimensional chemical shift imaging sequence (CSI_2D) in 17 of 18 patients and in all 10 control subjects, while in only one patient single-voxel spectroscopy (SVS) was acquired due to technical constraints at the time of scanning (patient motion during the longer CSI acquisition). The CSI sequence employed a spin-echo-based (PRESS-type) implementation with a slice-selective 90° excitation pulse for through-plane localization (15 mm thickness) and two-dimensional phase encoding using a weighted full k-space sampling strategy with a 16 × 16 matrix (256 phase-encoding steps) within a predefined volume of interest positioned in the periventricular white matter at the level of the centrum semiovale. This yielded a nominal voxel size of 10 × 10 × 15 mm^3^, with acquisition parameters of TR/TE = 1690/135 ms and 4 averages. The single SVS acquisition was performed as PRESS with a voxel size of 20 × 20 × 20 mm, TR/TE = 1500/135 ms and 192 averages.

Water suppression was achieved using weak CHESS (CHEmical Shift Selective) pulses with a suppression bandwidth of 35 Hz, optimized via an automated pre-scan adjustment. Outer volume suppression (OVS) was applied consistently in all subjects using six saturation bands to reduce extracranial lipid contamination. B0 shimming was standardized using an advanced vendor-supplied volumetric shim procedure (GRE field map-based) prior to acquisition, and the water linewidth (FWHM) was maintained below 15 Hz. A single TE of 135 ms was used to ensure methodological consistency, to take advantage of the well-resolved singlet resonances of NAA, Cho and Cr, to attenuate short-T2 macromolecule contributions, and to minimise total acquisition time, which is a relevant practical consideration in this paediatric population imaged under general anaesthesia.

Imaging data were reviewed in consensus by two experienced pediatric neuroradiologists. To ensure voxel placement reproducibility, all CSI volume placements were performed by a single neuroradiologist following a standardized anatomical protocol (periventricular white matter at the level of the centrum semiovale) and were then independently verified by a second senior neuroradiologist. No significant discrepancies were observed. Formal interobserver variability analysis was not performed, which may be considered a limitation.

### MRS data processing and quantification

To account for potential age-related variability, metabolite ratios rather than absolute concentrations were used. Metabolite ratios are considered more stable across developmental stages, minimizing confounding.

Initial visual quality assurance of every individual spectrum was performed before averaging; spectra showing spurious echoes in the FID, artefactual peaks, or dominating lipid peaks due to signal bleed within the CSI grid were rejected. Pre-processing was carried out in MATLAB using the FID-A toolbox (17) and consisted of the following sequential steps applied per subject: (i) eddy-current correction; (ii) exponential apodisation with 0.8 Hz line broadening; (iii) zero- and first-order phase correction; (iv) frequency alignment by spectral registration; and (v) signal averaging. For group-level analysis, subject-averaged spectra were further frequency-aligned and averaged.

MRS data were post-processed using LCModel (Linear Combination of Model spectra), version 6.3-1P (Provencher SW Inc., Oakville, Ontario, Canada). LCModel automatically quantifies *in vivo* proton MRS spectra by fitting the acquired signal to a linear combination of metabolite basis spectra. The basis set, kindly provided by S. Provencher, was simulated using the GAMMA spectral simulation tool (18) for TE = 135 ms at 64 MHz (1.5 T) and comprised 14 metabolites: Ala, Cr, PCr, Gln, Glu, GPC, PCh, Ins, Lac, NAA, NAAG, Scyllo, Cr-CH₂ and Gua. Lipid resonances at 2.0, 1.3 and 0.9 ppm together with macromolecule resonances at 2.0, 1.7, 1.4, 1.2 and 0.9 ppm were included as simulated single peaks (19). LCModel default soft-constraint priors were applied to the low-concentration metabolites (Asp, GABA, Glc, Scyllo, Tau) and the spectral fitting range was 4.0–0.2 ppm. In addition to major metabolite peaks (NAA, Cho, and Cr), the output provided composite measures including creatine (Cr + phosphocreatine), choline (glycerophosphocholine + phosphocholine), and N-acetyl-containing compounds (NAA + NAAG). These composite values enabled calculation of refined metabolite ratios such as (NAA + NAAG)/Cr and (NAA + NAAG)/Cho, although the primary diagnostic focus remained on NAA/Cr and NAA/Cho as these ratios are routinely calculated by any MRI vendor's software.

Importantly, at 1.5 T and TE = 135 ms the resonances of NAA and NAAG at ∼2.0 ppm overlap strongly and cannot be reliably separated. In our LCModel detailed output the off-diagonal correlation coefficient between fitted NAA and NAAG was strongly negative (always more negative than −0.7 across spectra in which NAA and NAAG were fitted as separate peaks; mean ≈ −0.92, representative subject −0.919; in the small number of cases in which LCModel did not separate NAAG from NAA and reported a NAAG concentration of 0.0, the corresponding off-diagonal correlation was 0.0), consistent with the recommendation in the LCModel manual that, where a pairwise correlation is more negative than −0.3, the metabolite pair should be considered jointly. The individually fitted NAA value should therefore be interpreted as an estimate of total NAA (tNAA = NAA + NAAG) dominated by NAA, and the diagnostic thresholds reported here apply to this tNAA-based ratio rather than to a pure NAA-only quantification. The composite ratios using the (NAA + NAAG) sum (Table 5) explicitly avoid this fitting instability and are therefore methodologically more robust at 1.5 T.

Spectral quality control was performed prospectively. The LCModel-reported SNR (defined as the ratio of the maximum spectrum-minus-baseline within the analysis window to twice the rms residuals) was required to be > 6, and the LCModel-reported metabolite linewidth (FWHM) was required to be < 0.1 ppm; spectra failing either threshold would have been excluded. Fitting reliability of individual metabolites was assessed using the LCModel %SD (Cramér–Rao lower bound equivalent), and a %SD < 25% was considered reliable. No spectra were rejected on these criteria in the present cohort.

For each subject, the LCModel-fitted concentrations of all individually fitted metabolites were summed (excluding LCModel-reported composite sums to avoid double counting) and each individual metabolite was expressed as a fraction of this total spectral area. This internal normalisation, performed at the level of the individual subject prior to group averaging, minimises scanner-, vendor- and acquisition-related scaling differences and facilitates cross-study comparability without requiring water-scaled absolute quantification. Partial volume correction (CSF segmentation or voxel tissue fractionation) was not performed; because metabolite ratios are reported and Cr is used as an internal reference, CSF contamination affects numerator and denominator approximately proportionally and is largely cancelled in the ratio. White-matter heterogeneity intrinsic to the leukodystrophic process is, however, not removed by ratioing and is acknowledged as a limitation.

### Statistical analysis

Continuous variables are reported as mean and median (interquartile range), while categorical variables are presented as frequencies and percentages. The normality of variable distributions was assessed using the Shapiro–Wilk test, which indicated non-normal distribution of the metabolite ratios. Consequently, group comparisons between patients with Canavan disease and controls were performed using the non-parametric Mann–Whitney U test.

Receiver operating characteristic (ROC) analysis was conducted to evaluate the diagnostic performance of each metabolite ratio. Optimal cut-off values were determined using Youden's index. Given the limited sample size of the cohort, these cut-off values should be considered preliminary and exploratory, and require validation in independent cohorts before potential clinical implementation. Additional exploratory thresholds were estimated using the midpoint between the first quartile (Q1) of the Canavan disease group and the third quartile (Q3) of the control group for NAA/Cr and NAA/Cho ratios, and conversely between the Q1 of controls and Q3 of the Canavan group for the Cho/Cr ratio.

To account for potential optimism resulting from model evaluation on the same dataset used for model development, bootstrap resampling with 10,000 iterations was performed. In each bootstrap sample, model performance was estimated and compared with its performance when applied to the original dataset, and the average optimism across all iterations was calculated. This value was subtracted from the apparent area under the curve (AUC) to obtain optimism-corrected AUC estimates with corresponding 95% confidence intervals. All statistical analyses were performed using R software (version 4.4.0), and *p*-values < 0.05 were considered statistically significant.

## Results

**Figure 1:**
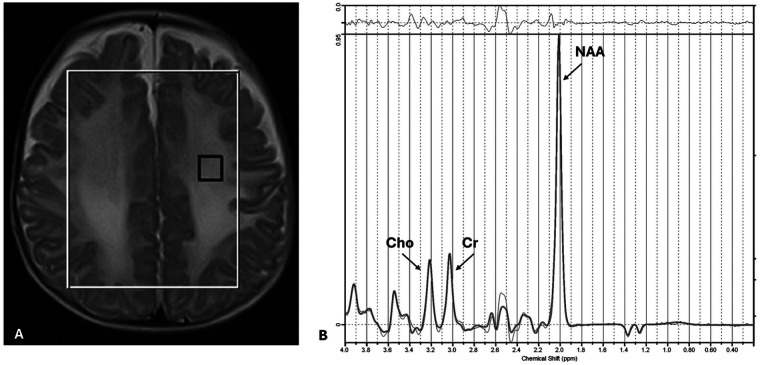
**(A)** axial T2-weighted MRI from a patient with canavan disease showing voxel placement in the left periventricular white matter for MRS acquisition. **(B)** Corresponding 1H-MRS spectrum demonstrates markedly elevated NAA peak, alongside Cr and Cho peaks, which are typically reduced in Canavan disease.

**Figure 2:**
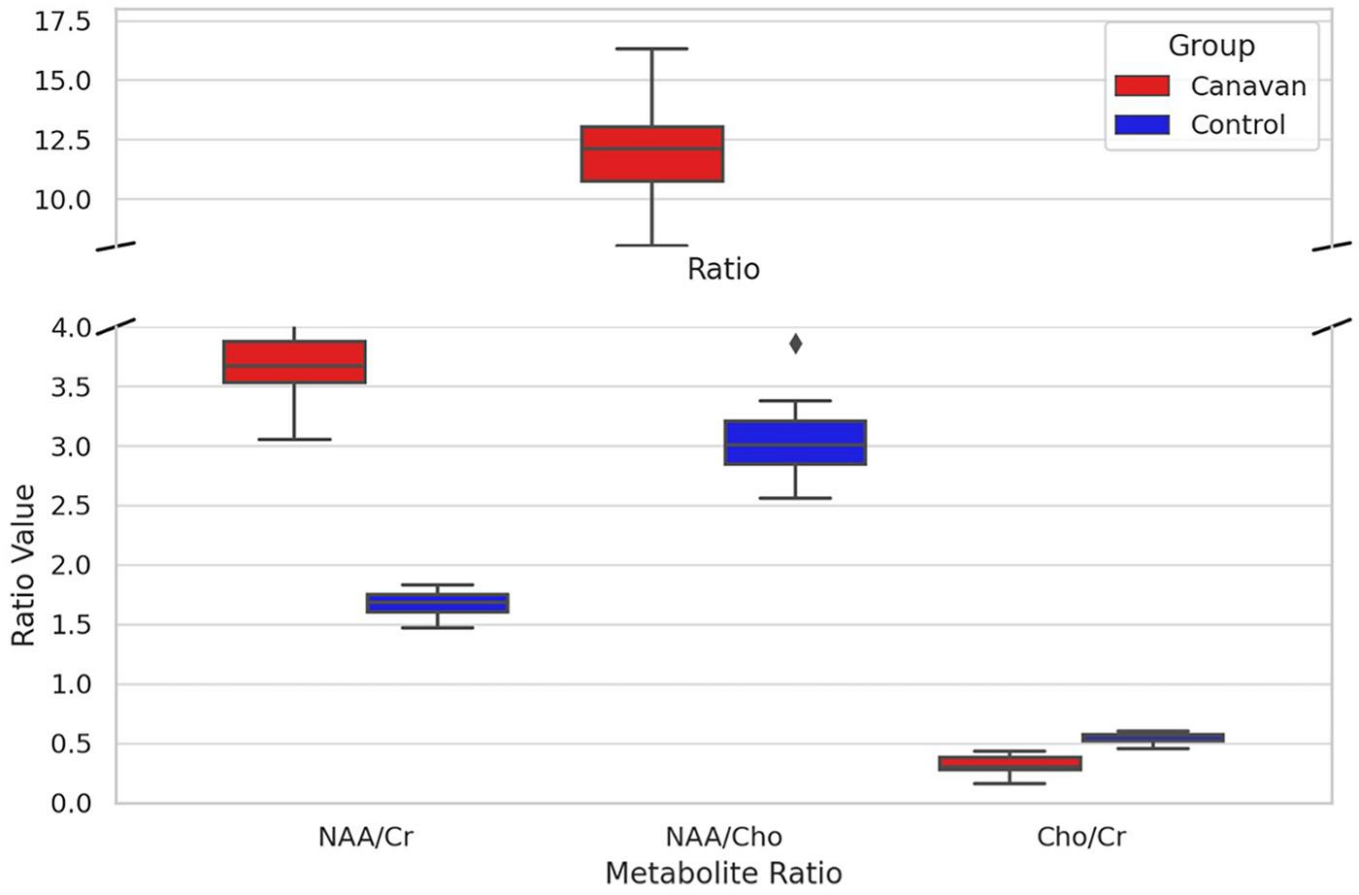
Representative images from a healthy control subject. **(A)** Axial T2-weighted MRI showing voxel placement in the left periventricular white matter. **(B)** Corresponding 1H-MRS output showing typical metabolite distribution in a normal brain, with visually assessed expected levels of NAA, Cr and Cho peaks.

Demographic characteristics are summarized in Table 1. There was no statistically significant difference in age between the Canavan and control groups (Mann–Whitney U, *p* = 0.854). Representative examples of voxel placement and corresponding 1H-MRS spectra are shown in Figures 1, 2.

**Table 1:** Demographic characteristics across groups.

Variable	Group	N	Mean	SD	Median	Q1	Q3
Age (months)	Control	10	9.8	6.14	10	5	12
	Canavan	18	16.6	24.24	7.5	6	16.75
Sex (M/F, n[%])	Control: 6/4 (60/40); Canavan: 9/9 (50/50)						

Mann–Whitney U comparison of age between groups: *p* = 0.854 (no significant difference).

**Figure 3:**
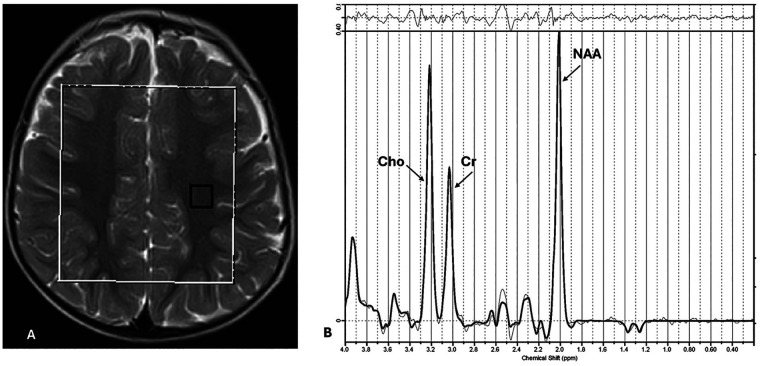
Box plots of NAA/Cr, NAA/Cho, and Cho/Cr ratios in CD (red) and control (blue) groups.

Table 2 presents the comparative analysis of metabolite profiles between the two groups, revealing statistically significant differences in metabolite concentrations (Figure 3). Notably, NAA/Cho demonstrated the most striking divergence, with the first quartile (Q1) of the CD group exceeding the third quartile (Q3) of the control group. This clear interquartile separation supports the potential discriminative utility of NAA/Cho within this cohort.

**Table 2:** Descriptive statistics, comparison of metabolite ratios and ROC curve analysis results between CD patients and control subjects.

Variable	Group	N	Mean	SD	Median	Q1	Q3	*p*-value*
NAA/Cr	Control	10	1.77	0.21	1.76	1.6	1.97	<0.001
	Canavan	18	3.92	0.51	3.79	3.58	4.07	
NAA/Cho	Control	10	3.27	0.43	3.34	2.89	3.48	<0.001
	Canavan	18	12.98	3.27	12.61	10.94	13.98	
Cho/Cr	Control	10	0.55	0.47	0.56	0.51	0.58	<0.001
	Canavan	18	0.32	0.73	0.30	0.30	0.38	

* Mann–Whitney U test.

For illustrative purposes, in a CD case (Figure 1), a markedly elevated NAA peak is evident, whereas Cr and Cho appear relatively reduced. In contrast, the spectrum from a healthy control (Figure 2) displays a typical metabolite distribution with balanced levels of NAA, Cr, and Cho.

As shown in Table 3, cut-off points for NAA/Cr and NAA/Cho with superior sensitivity and specificity can be determined. In the case of metabolite Cho/Cr, lower cut-offs show positive CD, unlike other metabolites.

**Table 3:** Diagnostic performance metrics for MRS metabolite ratios with optimism-corrected AUC (10,000 bootstrap iterations) and 95% CIs.

Variable	Optimism-corrected AUC	95% CI	Threshold
NAA/Cr	0.97	0.96–1.00	2.75
NAA/Cho	0.96	0.95–1.00	7.18
Cho/Cr	0.94	0.91–1.00	0.44

Given the limited sample size (18 Canavan, 10 controls) and the corresponding risk of overfitting, bootstrap resampling with 10,000 iterations was performed to obtain optimism-corrected AUC estimates with 95% confidence intervals. For NAA/Cr, the bootstrap-corrected AUC was 0.97 (95% CI 0.96–1.00); for NAA/Cho, the corrected AUC was 0.96 (95% CI 0.95–1.00). These values remained high after optimism correction, indicating that the apparent AUC of 1.00 was partially inflated by overfitting. Optimism-corrected AUCs for the LCModel-derived composite ratios are reported in Table 5. As a sensitivity analysis, all primary analyses were repeated after excluding the single SVS case; the results were virtually unchanged (NAA/Cr 3.21 vs. 3.19 in the full cohort; AUC remained 1.00 before, and identical after, bootstrap correction).

In addition to the broadly accessible MR vendor’s software ratios (NAA/Cr, NAA/Cho, Cho/Cr), we evaluated composite metabolite ratios derived from LCModel, including (NAA + NAAG)/Cr, (NAA + NAAG)/(Cr + PCr), (NAA + NAAG)/(GPC + PCh), NAA/(Cr + PCr), and (GPC + PCh)/Cr. These ratios incorporate phosphocreatine and NAAG components, which are automatically quantified by LCModel but may not be accessible in vendor-supplied MRS software. Furthermore, this analytical approach included normalization across subjects to the total spectral area, providing robust quantification ratios to establish reproducibility and cross-study comparability, thereby reducing the influence of age- and gender-related metabolic variability across subjects. ROC analysis revealed that several of these composite ratios, including (NAA + NAAG)/(Cr + PCr), (NAA + NAAG)/(GPC + PCh), and NAA/(Cr + PCr), achieved outstanding diagnostic performance (uncorrected AUC = 1.00; optimism-corrected AUC ≈ 0.95–0.97; Table 5). Therefore, they may provide promising discriminatory performance within this cohort and may represent potentially useful composite markers. Table 4 presents the diagnostic performance metrics for the MRS metabolite ratios, together with 95% confidence intervals estimated using 10,000 bootstrap iterations. Table 5 summarizes the AUC values and group means for these additional composite metrics.

**Table 4:** Diagnostic performance metrics for MRS metabolite ratios — 95% bootstrap confidence intervals for sensitivity, specificity, PPV, and NPV (10,000 iterations).

Variable	95% CI for sensitivity	95% CI for specificity	95% CI for PPV	95% CI for NPV
NAA/Cr	0.985–1.00	0.981–1.00	0.976–1.00	0.984–1.00
NAA/Cho	0.979–1.00	0.980–1.00	0.972–1.00	0.977–1.00
Cho/Cr	0.972–1.00	0.975–1.00	0.969–1.00	0.971–1.00

**Table 5:** ROC curve analysis of composite metabolite ratios derived from LCModel, with bootstrap-corrected AUC estimates (10,000 iterations).

Ratio	Bootstrap 95% CI	Optimism-corrected AUC
(NAA + NAAG)/(GPC + PCh)	0.956–1.000	0.968
(NAA + NAAG)/(Cr + PCr)	0.951–1.000	0.965
NAA/(Cr + PCr)	0.948–1.000	0.963
NAA/(GPC + PCh)	0.952–1.000	0.966
(NAA + NAAG)/Cr	0.932–1.000	0.953
NAA/Cr	0.930–1.000	0.952
(GPC + PCh)/(Cr + PCr)	0.000–0.089	0.006
(GPC + PCh)/Cr	0.000–0.094	0.008

## Discussion

1H-MRS in CD is characterized by a marked, isolated increase of the NAA peak, which has an important diagnostic value (9, 20–23). This study aimed to evaluate the feasibility of identifying potentially useful metabolite ratio cut-offs for Canavan disease, recognizing that these values should be considered preliminary and require further validation in independent cohorts. Three main metabolite ratios were measured in the white matter of the centrum semiovale, namely NAA/Cr, NAA/Cho, and Cho/Cr ratios, supplied by the MR vendor's software and after using a more accurate approach with third-party freely available software, which allowed for ratios’ normalization.

NAA is a marker of neuronal health and integrity. In normal development, NAA increases from birth to 6–8 months of age and becomes the dominant peak in the normal brain spectrum by 3–4 months of age, ceasing to rise significantly after early childhood (11, 21–27). This finding supports the use of MRS from periventricular white matter in CD patients for clinical diagnosis and for follow-up in future therapeutic studies (9, 26, 27).

Based on previous 1H-MRS studies in CD patients, NAA concentration rises to nearly 30% in comparison to the age-matched Control group (12, 20, 28, 29). In this study, all patients had a remarkable rise in NAA compared to the control group, with a mean value of 0.328 in CD patients versus 0.238 in controls, representing ∼37% increase. However, despite a rise in NAA being described as a pathognomonic finding, no validated cut-off values for metabolite ratios have been established in large CD patient cohorts using a single software analysis.

Cho and Cr also follow well-characterised developmental trajectories during the first three years of life, with smaller cohort studies in CD reporting reduced Cho and Cr compared with age-matched controls, consistent with impaired energy metabolism (9, 11, 12, 24, 26–32).

Moreover, we analyzed metabolite ratios rather than absolute concentrations, which are known to be less affected by age-related variability after infancy. Prior studies have demonstrated that NAA/Cr and NAA/Cho ratios stabilize beyond the first year of life, making them reliable for comparison across pediatric groups.

Reporting metabolite ratios rather than absolute concentrations is well established but introduces a dependency on the denominator. In CD, both Cr and Cho may be mildly reduced, which would tend to inflate the NAA-based ratios. This direction of bias makes the proposed diagnostic thresholds conservative for the inverse direction (i.e., more likely to misclassify a CD patient as a control rather than a control as a CD patient). Notwithstanding, total Cr (Cr + PCr) is widely accepted as the most stable internal reference in 1H-MRS of the brain and has been used in similar paediatric MRS studies (33, 34). To mitigate this concern further, we additionally report LCModel-derived composite ratios using the (Cr + PCr) and (GPC + PCh) sums as denominators (Table 5), which are less sensitive to fitting instability of the individual choline and creatine moieties.

In the study by Sarret et al. (2016), the NAA/Cho ratio in a patient with mild Canavan disease was 3–4 times higher than in age-matched controls, in whom NAA/Cho values in white matter typically range from 0.4 to 1.5 depending on age (35). Notably, this case illustrated that even in milder clinical presentations with less typical structural MRI findings, a markedly elevated NAA/Cho ratio can support the diagnosis when conventional imaging is inconclusive or subtle. Other studies have similarly reported increased NAA/Cr and NAA/Cho ratios in CD, although specific numerical values were often not provided (28, 36–38).

In our cohort of 18 CD cases, the mean NAA/Cho ratio was 12.98 versus 3.27 in controls. Given that Sarret et al. reported only one mild case, our series constitutes one of the largest published to date. Similarly, we observed a significant increase in the NAA/Cr ratio (3.92 vs. 1.77) and a reduction in the Cho/Cr ratio (0.32 vs. 0.55) when compared to control groups. These findings align with earlier studies suggesting elevated NAA and relatively suppressed choline levels in CD (9, 25, 39–41).

Based on our findings, an NAA/Cr ratio above 2.75 and an NAA/Cho ratio above 7.18 were suggestive of CD within this cohort, with high bootstrap-corrected diagnostic performance after optimism correction (NAA/Cr: optimism-corrected AUC = 0.97, 95% CI 0.96–1.00; NAA/Cho: optimism-corrected AUC = 0.96, 95% CI 0.95–1.00). These ratios demonstrated strong discriminatory potential in this cohort.

It should be noted that the diagnostic performance estimates reported herein are derived from comparisons with healthy controls and may overestimate accuracy in clinical settings where differentiation from other leukodystrophies or hypomyelinating disorders is required. Accordingly, the proposed metabolite ratio values should be considered provisional indicators rather than definitive diagnostic cut-offs.

Additionally, a Cho/Cr ratio below 0.44 yielded strong discriminatory power (AUC = 0.98), with a positive predictive value of 0.83 and a negative predictive value of 1.00. These metabolite ratio thresholds may serve as practical MRS-based criteria to complement conventional imaging in the diagnosis of CD.

LCModel provides metabolite ratios derived from fully processed spectra that were normalized to the total spectral area (the sum of all detectable metabolites). This internal normalization approach (referred to as all metabolites) minimizes variability introduced by baseline shifts, relaxation differences, and acquisition artifacts. Rather than relying on absolute quantification, which remains technically complex and less reproducible — ratio-based analysis using normalized data is now considered a robust and replicable method in MRS studies (29, 31). While NAA/Cr and NAA/Cho remain reliable diagnostic markers, our findings suggest that LCModel-derived composite ratios such as (NAA + NAAG)/(Cr + PCr) may offer enhanced diagnostic precision by capturing broader metabolic alterations (Table 5). However, their clinical use remains so far limited, though they are state-of-the-art in experimental and clinical brain research. Instead, standard MRI vendor platforms which typically report only combined signals (e.g., total NAA or Cr), restricting access to advanced metabolic biomarkers, are widely used. In our study, the advanced MRS analysis reveals that, in the presence of MRI findings confirming leukodystrophy, elevated levels in both ratios should be considered in clinical practice for accurate diagnosis, potentially aiding in early detection and treatment, particularly in differentiating CD from certain hypomyelination disorders that may present with increased NAA. Our proposed methodology might be preferable in establishing broadly used diagnostic metabolite ratios values rather than using sophisticated absolute metabolite normalization, to mitigate the effects caused by differences in data acquisition, which can be substantial and may impact data interpretation in multi-site/scanner studies using MRS (42).

This study has several limitations. The relatively small sample size (*n* = 18 patients, *n* = 10 controls) reflects the rarity of Canavan disease and limits both the statistical robustness of the diagnostic performance estimates and the generalisability of the findings beyond a single centre and a single 1.5 T MR scanner. Internal bootstrap correction (10,000 resamples) was therefore applied. The retrospective design introduces a potential selection bias, although genetic confirmation of all CD cases reduces the likelihood of diagnostic misclassification. Differences in age distribution between groups, as well as variability within the Canavan cohort, may represent confounding factors, given known age-related changes in metabolite levels in pediatric populations; although the use of metabolite ratios mitigates this effect, it does not eliminate it, and age-stratified analysis was not feasible due to the limited sample size. This imbalance partly reflects the limited availability of normal MRS data in very young infants in routine clinical practice. At 1.5 T and TE = 135 ms, NAA and NAAG cannot be reliably separated; the individually fitted NAA value should therefore be regarded as an estimate of total NAA (NAA + NAAG) and the proposed thresholds apply to this tNAA-based ratio. Both CSI (*n* = 17) and SVS (*n* = 1) acquisitions were used in the patient group; a sensitivity analysis excluding the single SVS case yielded virtually unchanged results, supporting the robustness of our findings. All examinations were performed under sedation or anaesthesia, as is standard in paediatric imaging; while no consistent clinically significant effects on major metabolite ratios have been reported (43), subtle influences cannot be entirely excluded. Finally, the absence of disease-control groups (other leukodystrophies and metabolic disorders) limits direct assessment of diagnostic specificity. The proposed thresholds should therefore be considered preliminary and require validation in larger, independent cohorts, ideally including disease mimics. As this study was conducted on a single-center 1.5 T scanner with a specific acquisition protocol, the proposed thresholds may vary across different field strengths, acquisition parameters, and vendor platforms.

Detailed clinical phenotype stratification (e.g., infantile versus juvenile forms) was not consistently available and may influence metabolite levels and threshold applicability.

## Conclusion

Our findings support the role of MRS in detecting the characteristic increase in NAA in Canavan disease and provide a quantitative framework for metabolite ratio analysis. This study demonstrates the feasibility of establishing diagnostic cut-off values using advanced MRS techniques and normalization approaches. However, the proposed thresholds should be considered preliminary and require validation in larger and independent cohorts, ideally including disease controls (other leukodystrophies and metabolic disorders), before clinical application.

## Data Availability

The original contributions presented in the study are included in the article/Supplementary Material, further inquiries can be directed to the corresponding author.
